# Simplification of Natural β-Carboline Alkaloids to Obtain Indole Derivatives as Potent Fungicides against Rice Sheath Blight

**DOI:** 10.3390/molecules25051189

**Published:** 2020-03-06

**Authors:** Jie Zeng, Zhijun Zhang, Qi Zhu, Zhiyan Jiang, Guohua Zhong

**Affiliations:** 1 Key Laboratory of Crop Integrated Pest Management in South China, Ministry of Agriculture and Rural Affairs, South China Agricultural University, Guangzhou 510642, China; zengjie@gdsyzx.edu.cn (J.Z.); zhuqi1008611@163.com (Q.Z.); zyjiang@stu.scau.edu.cn (Z.J.); 2 Key Laboratory of Natural Pesticide and Chemical Biology, Ministry of Education, South China Agricultural University, Guangzhou 510642, China; 3 School of Pharmacy, Lanzhou University, Lanzhou 730000, China

**Keywords:** *β*-carboline, indole, simplification, fungicidal activity, *Rhizoctonia solani*

## Abstract

The increasing resistance of rice sheath blight caused by *Rhizoctonia solani* highlights the need for highly effective and environmentally benign agents. Natural β-carboline alkaloids were simplified to obtain a series of indole derivatives, and their fungicidal activity and preliminary mode of action against *R. solani* were also evaluated. The initial hit indole (**7**) displayed significant fungicidal activity with an EC_50_ value of 25.56 μg/mL, and was selected for further optimization. Importantly, compound **55**, the most active compound, had an EC_50_ value of 0.62 μg/mL, and approximately 300-fold more potent than validamycin A (EC_50_ = 183.00 μg/mL). In vivo bioassay also demonstrated that compound **55** showed better fungicidal activities than validamycin A. Moreover, the mechanism studies revealed that compound **55** not only caused remarkable morphological and structural alterations of *R. solani* hyphae, but also induced the loss of mitochondrial membrane potential and interfered with DNA synthesis. Therefore, compound **55** showed superior fungicidal activity against *R. solani*, and the elucidated mode of action supported the potential application of compound **55** against rice sheath blight.

## 1. Introduction

Rice sheath blight, caused by *Rhizoctonia solani* Kühn, is one of the most serious rice diseases worldwide [1], and not only has led to devastating loss in rice production—up to 50% under favorable conditions—but also causes quality issues [2,3]. For example, in China, the sheath blight infects almost 18 million hectares of rice in 2016 [4]. Although fungicides such as validamycin [5] are currently the most effective and reliable method to manage this disease [6], pathogen resistance, residual toxicity and environmental problems may arise with constant and indiscriminate fungicide usage, which highlights the need for highly effective and environmentally benign agents to control rice sheath blight [7].

In the quest for novel fungicidal agents, natural bioactive products have been and remain an excellent source of novel structure and serve as templates and inspiration for fungicides [8,9]. However, natural bioactive products are often victims of several deficiencies such as paucity or difficulty of obtainment from natural sources, as some natural product leads possess rather large, complex, or labile chemical structures which are difficult to obtain quantitatively by chemical synthesis, and most are not optimally ideal for direct use because of these weak activities [10]. Therefore, structural modification or simplification of active lead compounds from natural products has proven to be a successful way to discover fungicides with new modes of action, such as strobilurins fungicides.

**Figure 1:**
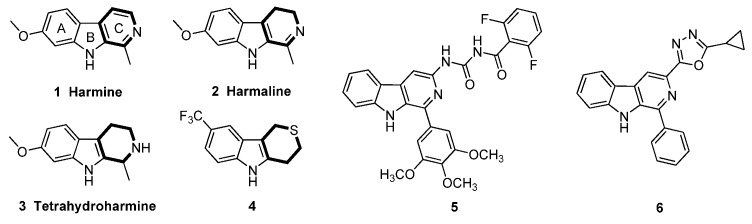
The structure of the *β*-carboline alkaloids and their derivatives.

Among natural product-based fungicides, β-carboline alkaloids and their saturated analogues, dihydro-β-carboline and tetrahydro-β-carboline alkaloids, isolated from medicinal genus *Peganum* [11], have possessed potent fungicidal activity with a broad spectrum [12,13,14]. For instant, the representative harmala alkaloids harmine (Figure 1, **1**), harmaline (**2**) and tetrahydroharmine (**3**) displayed marginal and similar fungicidal activity against *R. solani* [13]. In our previous work, we had introduced urea [15] and oxadiazole [16] groups into the 3 position of β-carboline scaffold and obtained two potent compounds **5** and **6** with respective EC_50_ values of 69.52 and 4.24 μg/mL against *R. solani*. Continued exploration of the β-carboline through optimization of the ring C of β-carboline led to 1,2,4,9-tetrahydro-3-thia-9-aza-fluorene derivatives with greater fungicidal efficacy giving rise to a highly effective agent **4** (EC_50_ = 2.35 μg/mL) [17], which indicated that the ring C of β-carboline was attractive for further simplifications. Therefore, in this study, we further simplified the β-carboline scaffold with an opening of ring C, and obtained a potent fungicidal template indole scaffold [18]. The structure-activity relationship (SAR) of indole compounds was then systematically evaluated and the preliminary mechanism of action was also elucidated.

## 2. Results and Discussion

### 2.1. Chemistry

The synthesis of intermediates and target compounds were performed as shown in the Supporting Information. N-substituted indole-2-carboxamides **11**–**14** were achieved in a good yield through coupling of indole-2-carboxylic acid and various amines with HCTU as a coupling reagent [19]. Indole-2-carboxylic acid was treated with diphenylphosphoryl azide (DPPA) to give the intermediate indole-2-carboxazide. Subsequently, rearrangment of this intermediate in various alcohols yielded the substituted indole-2-carbamates **15**–**18** [20]. Compound **19** was prepared according to our previously reported procedure [21]. *N*-benzylation of indole was accomplished using 2-chloro-5-chloromethylpyridine in the presence of sodium hydride, furnishing 1-(6-chloro-3-pyridylmethyl)-1H-indole **28**. Compounds **29**–**46** were prepared through acylation with various acyl chlorides under tetrabutylammonium hydrogen sulfate and potassium hydroxide [22]. Indole could be readily converted into indole-1-carboxylates **48**–**51** on treatment with various chloroformates, and into 1-(1*H*-indol-1-yl)-3-(4-chlorophenyl)urea **52** on treatment with 4-chlorophenyl isocyanate in the presence of sodium hydride [23]. Substituted indoles **55**, **56**, **58**, **60** and **74** on 4- or 5-position were synthesized from substituted 2-nitrotoluene with DMF-DMA through Leimgruber–Batcho procedure [24]. Likewise, *N*-benzoylation of aza-indoles and substituted indoles with 2-chlorobenzoyl chloride to obtain the corresponding derivatives **80**–**87**. All target compounds were purified by column chromatography and confirmed on the basis of ^1^H-NMR data.

### 2.2. Biological Activity

#### 2.2.1. In Vitro Fungicidal Activity against *R. Solani*

Firstly, a series of 2- or 3- position substituted indole derivatives were obtained through simplification of the β-carboline scaffold with an opening of ring C, and these ring C modified seco-structures **7**–**26** were screened for the in vitro fungicidal activity against *R. solani* and the results were shown in Table 1. Of these compounds, indole (**7**, EC_50_ = 25.56 μg/mL) displayed superior fungicidal activity, and was 7-fold as potent as validamycin A (EC_50_ = 183.00 μg/mL). Additionally, it can be seen quite clearly that the structural changes in general were detrimental to activity and no favorable substitution could be found for the 2- or 3-position, even after extensive probing with a variety of diverse groups (**8**–**26**). These findings implied that indole was a potent fungicidal template for further modification and derivatization.

The biological data for compounds **27**–**52** with a focus on varying substituents in the 1-position of the indole moiety were shown in Table 2. Here it can be seen that methyl (**27**) or very bulky 6-chloro-3-pyridylmethyl compound (**28**) displayed similar activity. Compounds **29**–**34** bearing linear alkyl groups showed similar potency with similar EC_50_ values. Interestingly, as the length of alkyl group increased, the activity of **29**–**34** correspondingly decreased at the concentration of 50 μg/mL, while the opposite results were obtained at the low concentration (10 μg/mL). In the 1-position, a cyclopropionyl (**35**) was favorable for activity, bulky cycloalkyl acyl groups (**36**–**38**) less so, but in contrast, good activity accompanied substitution with a range of benzoyl groups (**39**–**43**), particularly the decoration of the electron-withdrawing substituents on the ortho- position of benzoyl ring was recommended for the enhancement of activity than the meta- and para-counterparts (2-CF_3_ > 3-CF_3_ > 4-CF_3_). The fungicidal activity of ortho-substituted compounds (**40**–**43**) increased following 2-Cl ≈ 2-Br > 2-F > 2-CF_3_. For compounds **48**–**52**, the carboxylate substituents (**48**–**51**) was beneficial for the improvement of the fungicidal activity, while urea group (**52**) was detrimental to activity.

The SAR for the ring A of indole scaffold was probed next, and the results were depicted in Table 3. In the 4-position, compounds containing electron donating groups such as methyl (**53**) or methoxy group (**54**) retained fungicidal activity. For example, compound **53** (EC_50_ = 4.99 μg/mL) was over 5-fold more potent than indole (**7**, EC_50_ = 25.56 μg/mL). On the other hand, the introduction of a small electron withdrawing group (**55**–**60**) yielded highly active compounds, whereas a more polar derivative (**61**, 4-COOH) and a bulkier substituent (**62**, 4-COOCH_3_) showed inferior activity. Of particular note, compound **55** displayed the greatest inhibitory activity with the EC_50_ value of 0.62 μg/mL, and an over 40-fold higher activity than indole. In the 5-position, methyl (**63**), chloro (**66**), and bromo (**67**) were favorable for activity, a nitro (**68**) was tolerated, while the more polar (**69**–**71**) or bulky substituents (**74** and **75**) led to a loss of activity, which indicated that electronegativity and size of substituents were crucial for activity. A similar trend in activity was observed for compounds **76**–**79** bearing methyl or methoxy substitution at the 6- or 7-position of the indole ring.

**Figure 2:**
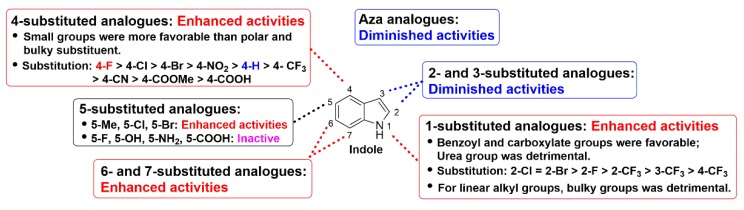
Detailed structure-activity relationships of indole compounds against *R. solani*.

The replacement of a CH group with a N atom in aromatic and heteroaromatic rings can have many important effects, such as molecular and physicochemical properties and intra- and inter-molecular interactions, which can translate to improved pharmacological profiles [25]. To evaluate whether a similar effect existed in indole ring, compounds **80**–**84** were synthesized and investigated (Table 4). Although these compounds displayed significant fungicidal activity against *R. solani*, with EC_50_ values ranging from 9.21–48.63 μg/mL, they were less potent than their corresponding counterpart **41** (EC_50_ = 2.15 μg/mL). According to the Table 1, Table 2, Table 3 and Table 4, we found that the compounds harboring 1-*O*-Cl-C_6_H_5_ (**41**), 4-F (**55**), 4-Cl (**56**), or 5-Cl (**66**), with respective EC_50_ values of 2.15, 0.62, 1.25 and 7.25 μg/mL, which were better than that of indole (25.56 μg/mL). Combined with these favorable substituents, compounds **85**–**87** resulted. Unfortunately, it can be seen that for compounds **85** and **86** this combination was detrimental to activity with respective EC_50_ values of 2.36 and 9.01 μg/mL as compared to compounds **55** and **56**, while a neutral effect was observed for compound **87**. The detailed structure-activity relationships of indole compounds were summarized in Figure 2.

#### 2.2.2. In Vivo Protective and Curative Effects against Rice Sheath Blight

**Figure 3:**
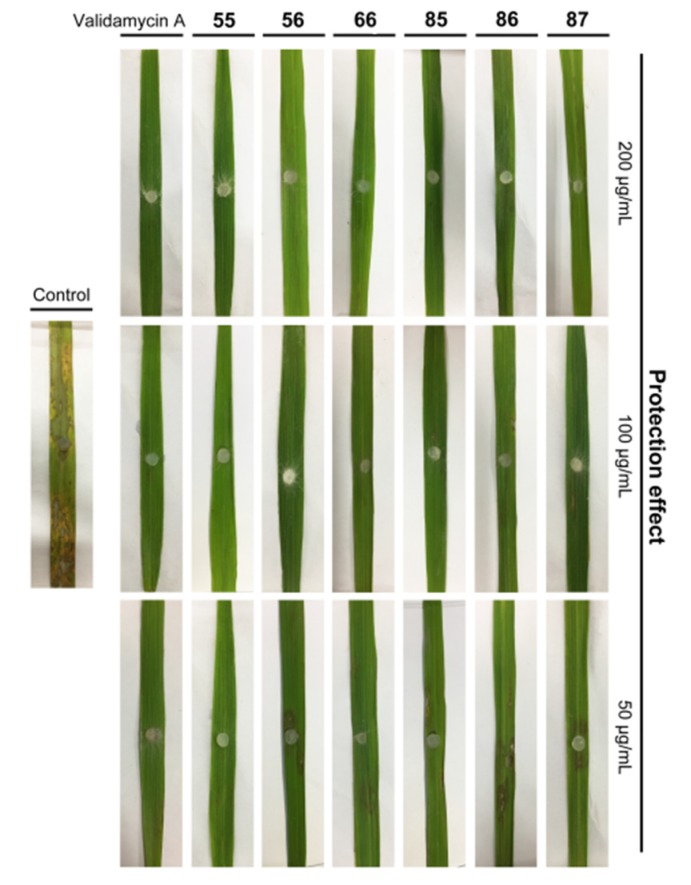
In vivo protective effect of selected compounds against *R. solani* using detached leaf assay.

Among the target compounds, compounds **55**, **56**, **66** and **85**–**87** displayed potent in vitro fungicidal activity against *R. solani* and, therefore, were selected to assess the in vivo protective effect through detached leaf assay. As shown in Table 5 and Figure 3, most of the tested compounds displayed excellent in vivo protective activity. To our delight, compound **55** possessed the highest fungicidal activity and the inhibitory effect at 50 μg/mL was up to 100%, which was comparable in activity to the commercial fungicide validamycin A (100% at 50 μg/mL).

**Figure 4:**
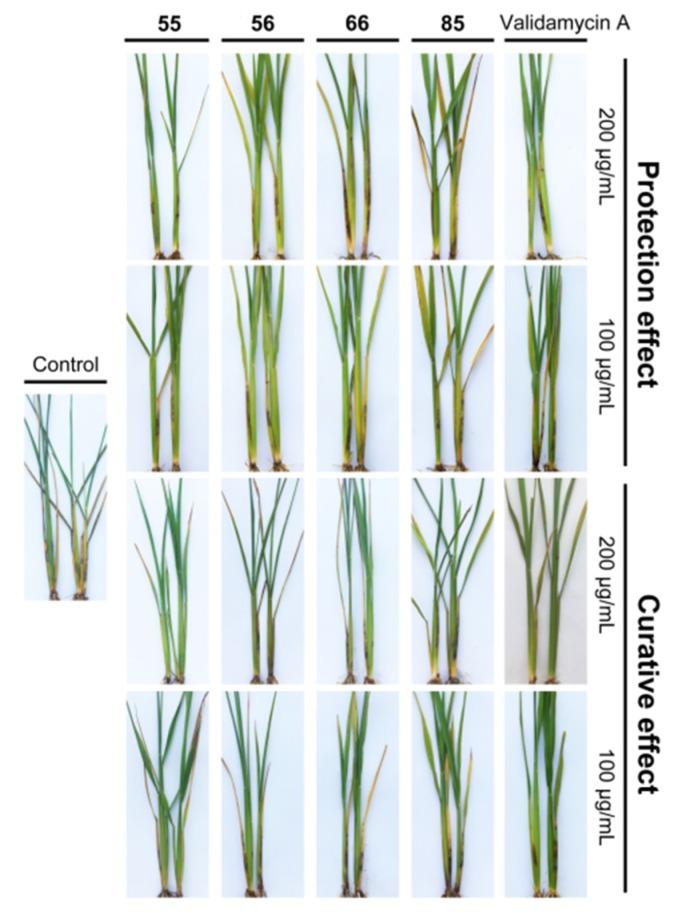
In vivo protective and curative activities of selected compounds against *R. solani.*

The most potent compounds **55**, **56**, **66** and **85** were further evaluated for their in vivo protective and curative activities against rice sheath blight in greenhouse experiment. As illustrated in Table 6 and Figure 4, most of these compounds displayed significant in vivo protective and curative activities against *R. solani*, of which compound **55** (79.23% and 89.08% at 200 μg/mL; 74.09% and 77.09% at 100 μg/mL) exhibited the highest fungicidal activity and was more potent than validamycin A (78.80% and 75.80% at 200 μg/mL; 65.74% and 67.45% at 100 μg/mL).

#### 2.2.3. Effect of **55** on the Hyphae Morphology of *R. Solani*

The inhibitory activity of compound **55** on the sclerotia germination and formation of *R. solani* was tested, and the results showed that compound **55** displayed strong inhibitory effects of sclerotia formation and germination in a dose-dependent manner and both the inhibitory rates reached 100% at the concentration of 10 μg/mL, which indicated that compound **55** could significantly inhibit sclerotia formation and germination of *R. solani*, thereby reducing the primary infection sources and controlling the disease (Figure S1).

### 2.3. Preliminary Mechanism of Compound **55** against R. Solani

#### 2.3.1. Effect of **55** on the Hyphae Morphology of *R. Solani*

The mycelia of *R. solani* grew smoothly along the surface of **55**-free medium with a low density and a regular colony. However, the mycelia was dense as compared to the control mycelia, and the edge of the colony was irregular in the presence of **55**, which implied that this compound could seriously depress the growth of mycelia.

**Figure 5:**
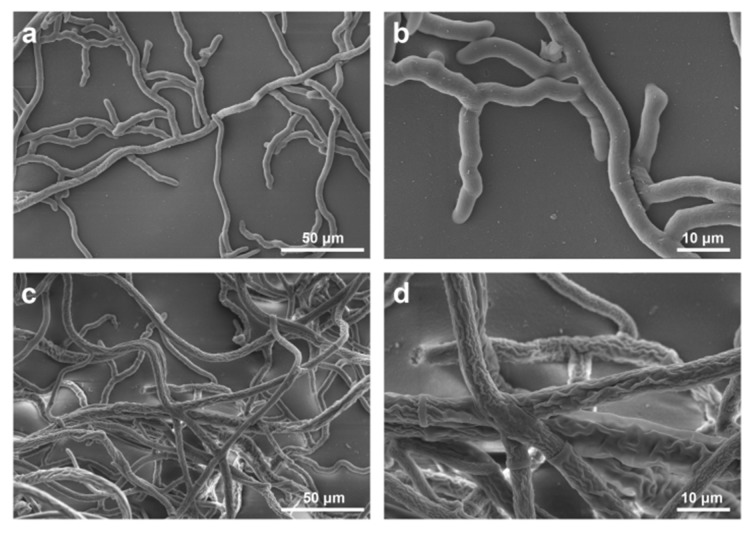
SEM of *R. solani* hyphae treated with 0 (**a**,**b**) or 1 μg/mL **55** (**c**,**d**).

The morphological variations of *R. solani* hyphae treated with 1 μg/mL **55** were revealed under SEM and the results were presented in Figure 5. The control mycelia were loose with a smooth surface and an intact structure (Figure 5a,b). However, the **55**-treated mycelia were dense with a coarse surface and locally folding and entangling, even appeared severely shrunken and distorted (Figure 5c,d).

**Figure 6:**
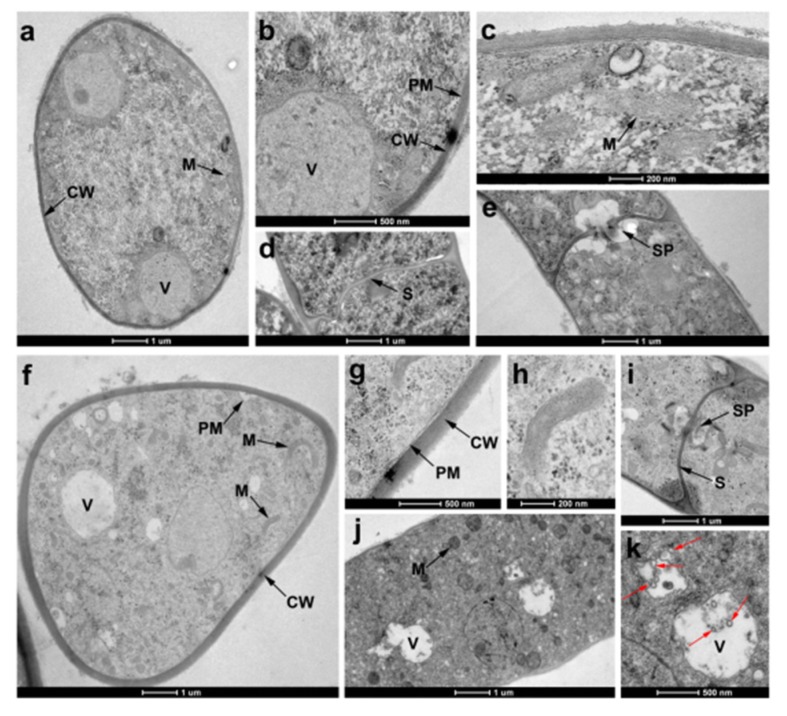
TEM of *R. solani* hyphae treated without (**a**–**e**) or with 1 μg/mL **55** (**f**–**k**). (**a**) transverse of untreated hyphae, and many organelles were observed such as mitochondria (M) and vacuole (V); (**b**) cell wall (CW) of untreated hyphae; (**c**) mitochondria of untreated hyphae; (**d**) longitudinal of untreated hyphae, and spectra (S) and was uniform; (**e**) septal pore caps (SP) of untreated hyphae was visible; (**f**,**g**) transverse of **55**-treated hyphae, and cell wall was thickening; (**h**) mitochondria of **55**-treated hyphae was swollen; (**i**) longitudinal of **55**-treated hyphae, and the septal pore caps disappeared; (**j**,**k**) longitudinal of **55**-treated hyphae (loss of matrix in vacuoles and obvious vacuolization), and red arrow represented vacuolization.

TEM was used to observe the definitive ultrastructural features of *R. solani* hyphae in response to compound **55**. As shown in Figure 6a–e, the control hyphal cell displayed the characteristic cellular features with obvious cell walls (CW), cell membranes (CM), septa (S) and septal pore caps (SP), and abundant organelles in cytoplasm, such as vacuole (V) and mitochondria (M). In contrast, the cell walls of the **55**-treated hyphae became abnormally thickened (Figure 6f,g). Moreover, the organelles were disorganized and caused abnormal changes. For instance, obvious cavitations and a slight swelling of mitochondrial matrix were observed after exposure to compound **55** (Figure 6h,j,k). It was notable that the septal pore caps of **55**-treated hyphae disappeared (Figure 6i).

#### 2.3.2. Effect of **55** on the Endogenous ROS Production and Cell Membrane Permeability

**Figure 7:**
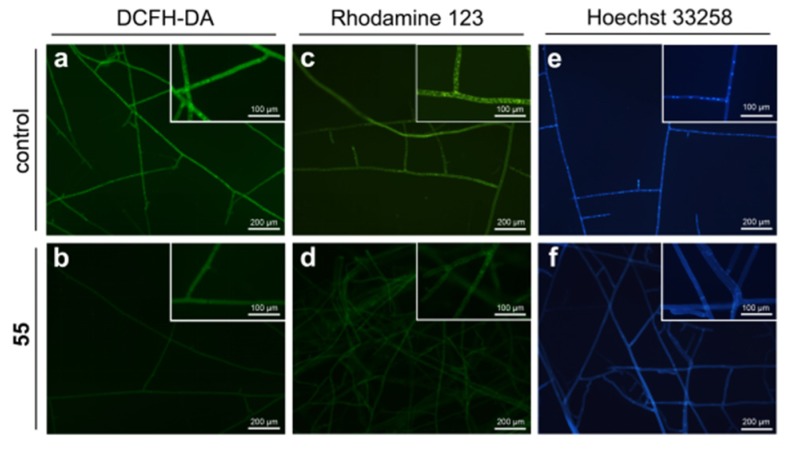
The mode of action of **55** against *R. solani*. (**a**,**b**) The intracellular ROS generation of the hyphae was detected by fluorescent micrographs using DCFH-DA staining; (**c**,**d**) The mitochondrial membrane potential of the hyphae was evaluated by fluorescent micrographs with Rhodamine 123 staining; (**e**,**f**) nuclear morphology of R. solani hyphae stained by Hoechst 33258.

Reactive oxygen species (ROS), byproducts of cellular metabolism, are primarily generated in the mitochondria. ROS accumulation could lead to cell damage and is considered as a fungicidal mechanism [26]. The effect of compound **55** on the endogenous ROS induction was determined by ROS assay using DCFH-DA staining. Surprisingly, the fluorescence intensity in the **55**-treated mycelia reduced obviously as compared to that in the control mycelia (Figure 7a,b), indicating that this compound inhibited the intracellular ROS generation. This result was opposed to our previous findings for β-carboline [16] and 1,2,4,9-tetrahydro-3-thia-9-aza-fluorene derivatives [17]. Next, we examined the effect of **55** on the cell membrane permeability through evaluating the conductivity alterations of the *R. solani* hyphae in response to **55**, and the results showed that compound **55** had no effect on cell membrane permeability (Figure S2a).

#### 2.3.3. Effect of **55** on the Mitochondrial Membrane Potential (MMP)

The effect of compound **55** on mitochondrial membrane potential (MMP) was detected using the potential-dependent distributional probe Rhodamine 123 and the results were presented in Figure 7c,d. The fluorescence intensity of **55**-treated hyphae dramatically decreased as compared to that of the control hyphae, which demonstrated that compound **55** could affect the MMP, disrupt the normal function of mitochondria, ultimately resulting in cell death.

#### 2.3.4. Effect of **55** on the Nuclear Morphology

Hoechst 33258 staining was utilized to observe the nuclear morphology of *R. solani* hyphae exposed to 55. The results showed that the cell nucleus of the control hyphal showed uniform blue color and were observed as intense, discrete signals. In contrast, the nucleus of 55-treated hyphae displayed faint signals, and most of which did not show discrete nuclear signals (Figure 7e,f). Moreover, the number of nuclei in per hyphal cell treated with 0 or 1 μg/mL 55 was determined by a quantitative analysis (Figure S2b), and the results showed that the number of nuclei in per 55-treated hyphal cell (average 3.67 nuclei) was fewer than that of per untreated hyphae cell (average 5.94 nuclei). Taken together, these findings suggested that compound 55 could reduce DNA contents and the number of cell nuclei of *R. solani* hyphae, thereby interfering with DNA synthesis.

## 3. Materials and Methods

### 3.1. Chemistry

All commercial reagents and solvents were of reagent grade and used without purification. The ^1^H-NMR spectral data were obtained using a Bruker Avance-600 superconducting nuclear magnetic resonance instrument (Bruker Company, Hamburg, Gemany). The synthesis and structural characterization of compounds **11**–**87** were showed in the Supporting Information.

### 3.2. Biological Assay

#### 3.2.1. In Vitro and in Vivo Fungicidal Activity against *R. Solani*

The fungi *R. solani* GD-118 was provided by our laboratory, and the rice cultivar (Xiangyazhan) was planted according to our previously reported method [15]. The in vitro and in vivo fungicidal activity against *R. solani* were tested following our previously reported method [17].

#### 3.2.2. Inhibition of Compound **55** on the Sclerotia Formation and Germination of *R. Solani*

The inhibitory activity of compound **55** on the sclerotia formation and germination of *R. solani* was evaluated using our previous method [17]. 

### 3.3. Morphology Observation of R. Solani Hyphae

The morphology changes of *R. solani* hyphae treated with 1 μg/mL compound **55** were detected under scanning electron microscopy (SEM) and transmission electron microscopy (TEM) using our previously reported method [16].

### 3.4. Reactive Oxygen Species (ROS) Generation

The *R. solani* hyphae were treated with 0 and 1 μg/mL compound **55** for 48 h, and then were cut and placed on a sterile slide before continued incubation at 25 °C for 24 h. The hyphae were stained with 10 μM DCFH-DA solution (Beyotime, Shanghai, China). After incubation at 37 °C for 30 min in the darkness, the samples were observed and photographed using a fluorescence microscopy (Nikon ECLIPES 80 *i*, Tokyo, Japan).

### 3.5. Mitochondrial Membrane Potential (MMP)

Rhodamine 123 was used as an indicator to detect the effect of **55** on the MMP of *R. solani* using our previously reported method [16]. 

### 3.6. Karyological Analysis

The mycelial specimens were fixed with stain-fixative at 4 °C overnight. After washing twice with 0.1 M PBS, the fixed hyphae were stained with 1 mL Hoechst 33258 solution (Beyotime) at 25 °C for 10 min and the samples were observed using a fluorescence microscopy.

### 3.7. Detection of Cell Membrane Permeability 

The effect of compound **55** on the cell membrane permeability was evaluated through the conductivity changes of *R. solani* mycelial using our previously reported method [16].

## 4. Conclusions

Simplification of β-carboline alkaloids through an opening of ring C obtained a potent fungicidal template indole scaffold. The structure-activity relationship (SAR) of indole compounds was then systematically evaluated and the preliminary mode of action of compound **55** was also elucidated. Among these compounds, compound **55** showed the most active against *R. solani* with an EC_50_ value of 0.62 μg/mL, and was approximately 300-fold more potent than validamycin A. In vivo bioassay also demonstrated that compound **55** displayed superior protective and curative activities as compared to validamycin A. Moreover, the preliminary mechanism studies revealed that compound **55** caused remarkable morphological and structural alterations of *R. solani* hyphae, induced the loss of mitochondrial membrane potential and reduced the DNA contents and number of cell nuclei. Due to these positive results, further development of **55**-related compounds as potential fungicidal candidates is definitely warranted.

## Figures and Tables

**Table 1:**
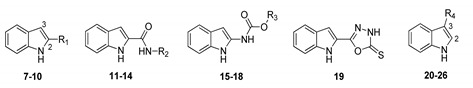
In vitro fungicidal activity of compounds **7**–**26**. ^a^

Compound	R	Percentage Inhibition (%)		EC_50_ (95%CI ^c^) (μg/mL)
		50 μg/mL	10 μg/mL	
Indole (**7**)	H	87.68 ± 1.26	26.33 ± 3.64	25.56 (23.61–27.66)
**8**	CH_3_	28.65 ± 1.69	12.47 ± 2.21	- ^d^
**9**	Ph	25.45 ± 5.45	10.67 ± 1.79	-
**10**	COOCH_3_	26.04 ± 1.54	9.35 ± 1.33	-
**11**	cyclopropyl	29.21 ± 1.63	22.47 ± 2.33	58.35 (48.45–70.55)
**12**	morpholine	32.79 ± 3.36	2.15 ± 2.46	92.19 (78.24–114.69)
**13**	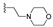	20.97 ± 3.22	0.00	-
**14**	4-Cl-Ph	51.35 ± 3.57	40.22 ± 0.48	52.84 (39.46–70.83)
**15**	Me	55.81 ± 0.74	36.08 ± 2.68	51.96 (39.78–65.79)
**16**	Et	57.53 ± 3.64	35.01 ± 0.91	53.20 (39.11–68.64)
**17** ^ b^	*^i^*Pr	56.88 ± 0.91	32.43 ± 2.73	53.43 (43.14–65.59)
**18**	*^n^*Bu	44.23 ± 1.97	36.51 ± 1.97	77.79 (55.81–128.22)
**19**	-	54.05 ± 1.35	20.27 ± 1.35	60.22 (51.18–71.11)
**20**	Ac	56.18 ± 1.79	24.94 ± 1.79	
**21**	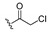	71.75 ± 4.26	27.38 ± 2.73	40.74 (26.89–54.89)
indole-3-carboxylic acid (**22**)		20.18 ± 1.79	15.95 ± 1.79	-
indole-3-carbinol (**23**)		30.67 ± 1.79	10.45 ± 1.79	-
indole-3-acetic acid (**24**)		44.94 ± 1.79	2.25 ± 1.79	-
*L*-tryptophan (**25**)		33.71 ± 1.79	22.47 ± 1.79	-
Gramine (**26**)		0.00	0.00	-
validamycin A		36.68 ± 1.09	18.91 ± 0.49	183.00 (162.62–210.66)

^a^ Values are the mean ± SD of three replicates. ^b^ novel compounds. ^c^ 95% confidence interval. ^d^ not determined.

**Table 2:**
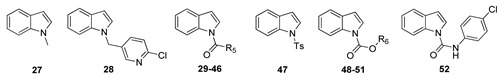
In vitro fungicidal activity of compounds **27–52**. ^a^

Compound	R	Percentage Inhibition (%)		EC50 (95%CI ^c^) (μg/mL)
		50 μg/mL	10 μg/mL	
**27**		90.84 ± 8.54	5.57 ± 2.44	32.82 (29.22–36.55)
**28** ^ b^		73.24 ± 4.39	40.66 ± 1.74	24.07 (20.57–27.78)
**29**	Me	89.54 ± 2.47	34.44 ± 3.69	21.94 (19.86–24.11)
**30**	Et	93.55 ± 0.00	27.42 ± 1.61	22.97 (21.11–24.95)
**31**	*^n^*Pr	88.09 ± 1.03	33.33 ± 5.45	28.25 (13.28–40.70)
**32**	*^n^*Bu	84.52 ± 1.03	41.07 ± 1.78	21.39 (12.27–29.28)
**33**	*n*-pentyl	72.60 ± 1.29	42.26 ± 1.03	31.25 (16.38–44.19)
**34** ^ b^	*n*-hexyl	63.98 ± 0.93	32.26 ± 4.84	45.71 (34.83–57.55)
**35**	cyclopropyl	87.63 ± 1.86	55.38 ± 1.86	17.47 (9.85–24.93)
**36** ^ b^	cyclobutyl	76.88 ± 0.93	34.95 ± 2.46	29.93 (21.62–37.83)
**37** ^ b^	cyclopentyl	76.19 ± 2.73	29.38 ± 1.96	35.21 (24.04–46.05)
**38**	cyclohexyl	71.75 ± 0.98	41.24 ± 5.1	28.25 (13.28–40.70)
**39**	Ph	79.74 ± 4.08	57.43 ± 1.95	8.80 (2.29–14.39)
**40**	2-F-Ph	87.20 ± 1.01	66.26 ± 1.01	3.53 (0.91–8.54)
**41**	2-Cl-Ph	81.38 ± 1.01	71.90 ± 2.55	2.15 (0.54–6.01)
**42**	2-Br-Ph	79.06 ± 1.74	69.77 ± 0.74	2.74 (0.51–7.79)
**43** ^ b^	2-CF_3_-Ph	74.15 ± 1.10	64.74 ± 0.84	9.25 (4.65–14.06)
**44**	3-CF_3_-Ph	71.05 ± 0.93	51.61 ± 1.61	17.81 (2.34–29.25)
**45**	4-CF_3_-Ph	40.68 ± 1.69	34.74 ± 1.20	85.40 (65.26–127.37)
**46** ^ b^	4-OCF_3_-Ph	38.42 ± 3.53	26.19 ± 1.03	89.06 (77.04–106.52)
**47**		69.17 ± 2.66	52.30 ± 1.01	17.70 (7.67–26.35)
**48**	Me	100.00	75.22 ± 0.78	4.12 (2.01–5.28)
**49**	Et	79.73 ± 1.35	53.60 ± 4.74	12.68 (7.37–17.31)
**50**	*^i^*Pr	80.18 ± 1.56	59.01 ± 4.34	6.87 (1.00–13.49)
**51**	*^t^*Bu	80.80 ± 1.74	58.11 ± 1.74	17.87 (2.75–31.36)
**52**		30.11 ± 1.86	24.73 ± 1.86	129.48 (91.93–278.45)

^a^ Values are the mean ± SD of three replicates. ^b^ novel compound. ^c^ 95% confidence interval.

**Table 3:**
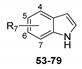
In vitro fungicidal activity of compounds **53–79**. ^a^

Compound	R	Percentage Inhibition (%)		EC_50_ (95%CI ^b^) (μg/mL)
		50 μg/mL	10 μg/mL	
**53**	4-CH_3_	100	76.80±3.15	4.99 (4.46–5.50)
**54**	4-OCH_3_	87.23 ± 2.21	50.56 ± 1.33	25.30 (16.51–35.74)
**55**	4-F	**100**	**100**	**0.62 (0.53–0.69)**
**56**	4-Cl	**100**	**100**	**1.25 (0.97–1.51)**
**57**	4-Br	100	58.65 ± 1.93	7.96 (6.99–9.07)
**58**	4-NO_2_	100	18.99 ± 2.33	16.13 (4.45–30.55)
**59**	4-CN	93.26 ± 1.37	6.32 ± 1.71	45.50 (26.42–81.96)
**60**	4-CF_3_	100	8.86 ± 0.89	38.58 (25.45–51.97)
**61**	4-COOH	34.83 ± 1.21	6.32 ± 1.01	66.89 (38.42–94.17)
**62**	4-COOCH_3_	64.05 ± 6.38	0.00	46.76 (21.08–79.48)
**63**	5-CH_3_	100	37.44 ± 3.67	11.83 (10.84–13.40)
**64**	5-OCH_3_	54.90 ± 4.88	0.00	48.10 (44.97–51.55)
**65**	5-F	1.15 ± 0.00	0.00	- ^c^
**66**	5-Cl	100	59.17 ± 8.07	7.25 (5.92–8.48)
**67**	5-Br	100	49.86 ± 0.72	7.94 (6.34–9.50)
**68**	5-NO_2_	97.37 ± 4.55	10.22 ± 2.19	26.78 (23.47–30.66)
**69**	5-OH	0.00	0.00	-
**70**	5-NH_2_	0.00	0.00	-
**71**	5-COOH	33.05 ± 8.00	0.00	61.92 (52.62–79.08)
**72**	5-CHO	59.13 ± 3.23	0.00	45.37 (41.26–50.23)
**73**	5-CN	73.97 ± 1.09	1.15 ± 0.00	40.72 (38.75–42.68)
**74**	5-Ac	9.09 ± 3.66	0.00	-
**75**	5-OBz	42.92 ± 3.66	0.00	53.03 (50.55–56.02)
**76**	6-OCH_3_	47.64 ± 3.38	34.83 ± 1.47	-
**77**	6-Cl	100	32.35 ± 2.11	12.36 (11.35–13.98)
**78**	7-OCH_3_	84.25 ± 0.74	46.07 ± 0.61	28.03 (19.85–37.56)
**79**	7-Cl	100	53.49 ± 4.61	9.71 (9.11–10.47)

^a^ Values are the mean ± SD of three replicates. ^b^ 95% confidence interval. ^c^ not determined.

**Table 4:**
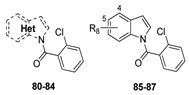
In vitro fungicidal activity of compounds **80–87**. ^a^

Compound	R	Percentage Inhibition (%)		EC_50_ (95%CI ^c^) (μg/mL)
		50 μg/mL	10 μg/mL	
**80** ^ b^	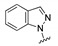	80.00 ± 1.21	40.45 ± 0.72	25.32 (13.57–40.45)
**81**	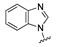	83.15 ± 3.66	53.93 ± 1.97	9.21 (1.57–21.02)
**82** ^ b^	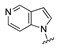	78.65 ± 0.38	49.44 ± 1.09	19.31 (4.96–32.77)
**83** ^ b^	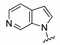	56.18 ± 1.69	22.47 ± 1.79	48.63 (40.42–57.72)
**84** ^ b^	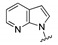	71.91 ± 3.27	44.94 ± 1.33	29.52 (11.04–59.41)
**85** ^ b^	4-F	72.03 ± 2.10	63.32 ± 0.79	2.36 (0.23–8.25)
**86** ^ b^	4-Cl	69.28 ± 0.79	61.48 ± 0.00	9.01 (2.85–19.81)
**87** ^ b^	5-Cl	70.04 ± 0.73	65.82 ± 0.00	5.31 (3.08–13.34)

^a^ Values are the mean ± SD of three replicates. ^b^ 95% confidence interval.

**Table 5:** In vivo protective activity against *R. solani* using detached leaf assay.

Treatment	Concentration (μg/mL)	Lesion Length ^a^ (cm ± SE)	Control Efficacy (%)
**55**	200	**0** **	**100**
	100	**0** **	**100**
	50	**0** **	**100**
**56**	200	**0** **	**100**
	100	**0** **	**100**
	50	1.65 ± 1.20 **	79.50
**66**	200	**0** **	**100**
	100	**0** **	**100**
	50	0.43 ± 0.34 **	94.66
**85**	200	**0** **	**100**
	100	0.11 ± 0.12 **	98.63
	50	1.07 ± 0.67 **	86.71
**86**	200	0.72 ± 0.49 **	91.05
	100	1.80 ± 0.84 **	77.64
	50	2.41 ± 0.89 **	70.19
**87**	200	0.15 ± 0.23 **	98.14
	100	0.55 ± 0.35 **	93.17
	50	1.17 ± 0.91 **	87.95
validamycin A	200	0 **	100
	100	0 **	100
	50	0 **	100
control	0	8.05 ± 1.01	

** represents *P* < 0.01. ^a^ Values are the mean ± SD of 20 leaves.

**Table 6:** In vivo protective and curative effects against *R. solani* using greenhouse experiment.

Treatment	Conc. (μg/mL)	Protective Effect		Curative Effect	
		Lesion Length ^a^ (cm ± SE)	Control Efficacy (%)	Lesion Length (cm ± SE)	Control Efficacy (%)
**55**	200	**0.97 ± 0.31** **	**79.23**	**0.51 ± 0.36** **	**89.08**
	100	**1.21 ± 0.70** **	**74.09**	**1.07 ± 0.46** **	**77.09**
**56**	200	1.05 ± 0.41 **	77.52	**0.98 ± 0.40** **	**79.01**
**66**	200	**1.01 ± 0.68** **	**78.37**	1.15 ± 0.36 **	75.37
	100	**1.51 ± 0.61** **	**67.66**	1.48 ± 0.42 **	**68.31**
**85**	200	1.39 ± 0.71 **	70.23	1.95 ± 0.35 **	58.24
	100	2.06 ± 0.56 **	55.89	3.62 ± 0.50 **	22.48
validamycin A	200	0.99 ± 0.43 **	78.80	1.13 ± 0.51 **	75.80
	100	1.60 ± 0.60 **	65.74	1.52 ± 0.64 **	67.45
control	0	4.67 ± 0.97		4.67 ± 0.97	

** represents *P* < 0.01. ^a^ Values are the mean ± SD of 20 plants.

## Supplementary Materials

Supplementary Materials are available online.

## References

[1] Groth D.E. (2005). Azoxystrobin rate and timing effects on rice sheath blight incidence and severity and rice grain and milling yields. Plant Dis..

[2] Lee F.N., Rush M.C. (1983). Rice sheath blight: A major rice disease. Plant Dis..

[3] Marchetti M.A. (1983). Potential impact of sheath blight on yield and milling quality of short-statured rice lines in the Southern United States. Plant Dis..

[4] Xiang Y., Zhang Y., Wang C., Liu S., Liao X. (2018). Effects and inhibition mechanism of phenazine-1-carboxamide on the mycelial morphology and ultrastructure of *Rhizoctonia solani*. Pestic. Biochem. Phys..

[5] Boukaew S., Klinmanee C., Prasertsan P. (2013). Potential for the integration of biological and chemical control of sheath blight disease caused by *Rhizoctonia solani* on rice. World J. Microb. Biot..

[6] Mew T.W. (2004). Applying rice seed-associated antagonistic bacteria to manage rice sheath blight in developing countries. Plant Dis..

[7] Zhang C.Q., Liu Y.H., Ma X.Y., Feng Z., Ma Z.H. (2009). Characterization of sensitivity of *Rhizoctonia solani*, causing rice sheath blight, to mepronil and boscalid. Crop Prot..

[8] Isman M.B. (2006). Botanical insecticides, deterrents, and repellents in modern agriculture and an increasingly regulated world. Annu. Rev. Entomol..

[9] Rosell G., Quero C., Coll J., Guerrero A. (2008). Biorational insecticides in pest management. J. Pestic. Sci..

[10] Ichikawa S. (2016). Function-Oriented Synthesis: How to design simplified analogues of antibacterial nucleoside natural products?. Chem. Rec..

[11] Kartal M., Altun M.L., Kurucu S. (2003). HPLC method for the analysis of harmol, harmalol, harmine and harmaline in the seeds of *Peganum harmala* L.. J. Pharmaceut. Biomed..

[12] Song H., Liu Y., Liu Y., Wang L., Wang Q. (2014). Synthesis and antiviral and fungicidal activity evaluation of β-carboline, dihydro-β-carboline, tetrahydro-β-carboline alkaloids, and their derivatives. J. Agric. Food Chem..

[13] Li Z.B., Chen S.H., Zhu S.W., Luo J.J., Zhang Y.M., Weng Q.F. (2015). Synthesis and fungicidal activity of *β*-carboline alkaloids and their derivatives. Molecules.

[14] Khan H., Mubarak M.S., Amin S. (2017). Antifungal potential of alkaloids as an emerging therapeutic target. Curr. Drug Targets.

[15] Zhang Z.J., Zeng Y., Jiang Z.Y., Shu B.S., Sethuraman V., Zhong G.H. (2018). Design, synthesis, fungicidal property and QSAR studies of novel β-carbolines containing urea, benzoylthiourea and benzoylurea for the control of rice sheath blight. Pest Manag. Sci..

[16] Zhang Z.J., Jiang Z.Y., Zhu Q., Zhong G.H. (2018). Discovery of β-carboline oxadiazole derivatives as fungicidal agents against rice sheath blight. J. Agric. Food Chem..

[17] Xi J.M., Zhang Z.J., Zhu Q., Zhong G.H. (2018). Evolution from natural β-carboline alkaloids to obtain 1,2,4,9-tetrahydro-3-thia-9-aza-fluorene derivatives as potent fungicidal agents against *Rhizoctonia solani*. Int. J. Mol. Sci..

[18] Singh T.P., Singh O.M. (2018). Recent progress in biological activities of indole and indole alkaloids. Mini-Rev. Med. Chem..

[19] Mistry S.N., Shonberg J., Draperjoyce C.J., Klein H.C., Michino M., Shi L., Christopoulos A., Capuano B., Scammells P.J., Lane J.R. (2015). Discovery of a novel class of negative allosteric modulator of the dopamine D2 receptor through fragmentation of a bitopic ligand. J. Med. Chem..

[20] Jiang H., Zhuang D.M., Huang Y., Cao X., Yao J., Li J., Wang J.Y., Zhang C., Jiang B. (2014). Design, synthesis, and biological evaluation of novel trifluoromethyl indole derivatives as potent HIV-1 NNRTIs with an improved drug resistance profile. Org. Biomol. Chem..

[21] Zhang Z.J., Zhang J.J., Jiang Z.Y., Zhong G.H. (2017). Design, synthesis and bioactivity evaluation of novel β-carboline 1,3,4-oxadiazole derivatives. Molecules.

[22] Tomakinian T., Guillot R., Kouklovsky C., Vincent G. (2014). Direct oxidative coupling of *N*-acetyl indoles and phenols for the synthesis of benzofuroindolines related to phalarine. Angew Chem. Int. Edit..

[23] Kirchberg S., Fröhlich R., Studer A. (2009). Stereoselective palladium-catalyzed carboaminoxylations of indoles with arylboronic acids and TEMPO. Angew Chem. Int. Edit..

[24] Batcho A.D., Leimgruber W. (1985). Indoles from 2-methylnitrobenzenes by condensation with formamide acetals followed by reduction: 4-benzyloxyindole. Org. Synth..

[25] Pennington L.D., Moustakas D.T. (2017). The necessary nitrogen atom: A versatile high-impact design element for multiparameter optimization. J. Med. Chem..

[26] Hwang J.H., Hwang I.S., Liu Q.H., Woo E.R., Lee D.G. (2012). (+)-Medioresinol leads to intracellular ROS accumulation and mitochondria-mediated apoptotic cell death in *Candida albicans*. Biochimie.

