# Supplementary material for: Simplification of Natural β-Carboline Alkaloids to Obtain Indole Derivatives as Potent Fungicides against Rice Sheath Blight
Source: Molecules. 2020 Mar 6;25(5):1189. doi: 10.3390/molecules25051189 (PMC7179428; doi:10.3390/molecules25051189)
Supplement: Supplementary file 1 [file molecules-25-01189-s001.pdf]

Supplementary Data

Simplification of Natural *β*-Carboline Alkaloids to Obtain Indole Derivatives as Potent Fungicides against Rice Sheath Blight

Jie Zeng[1,2,†], Zhijun Zhang[1,2,3,†,*], Qi Zhu[1,2], Zhiyan Jiang[1,2], Guohua Zhong[1,2,*]

1. General

Compounds **7~10**, **20**, **22~27**, **53**, **54**, **57**, **59**, **61~73**, **75~79** were purchased from BePharm Co., Ltd. (Shanghai, China). All other reagents and solvents used in the study were analytical grade and obtained from commercial sources.

2. Chemistry

2.1 General procedure for the synthesis of compounds 11~14 [1]. To a solution of indole-2-carboxylic acid (161 mg, 1 mmol) and HCTU (455 mg, 1.1 mmol) in 5 mL DMF was added amine (3 mmol). The mixture was stirred at room temperature overnight and then diluted with water. After filtration, the resultant precipitate was collected and washed with water to obtain the desired compound **11~14**.

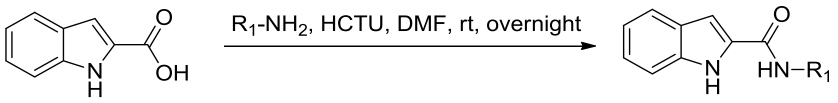

Indole-2-carboxylic acid **11-14**

2.2 General procedure for the synthesis of compounds 15~18. Indole-2-carboxylic

acid (161 mg, 1 mmol) and triethylamine (278 μL, 2 mmol) were dissolved in dichloromethane (10 mL) with stirring at room temperature. To this was added diphenylphosphoryl azide (DPPA, 275 mg, 1 mmol) and the mixture was stirred for 1 h and then concentrated to give the intermediate indole-2-carboxazide. The intermediate was dissolved in various alcohols and then was heated under reflux for 6 h. After concentration, the products **15~18** were obtained by column chromatography.

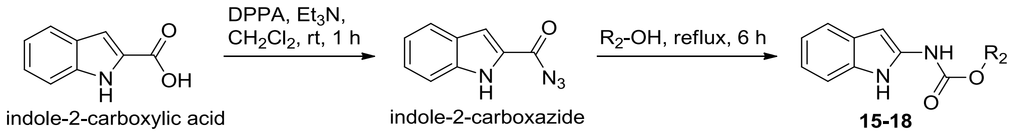

2.3 Synthesis of compound 19. To a solution of methyl indole-2-carboxylate (350 mg, 2 mmol) in 10 mL absolute ethanol was added 2 mL hydrazine hydrate and the mixture was heated under reflux for 6 h. The reaction mixture was then cooled, and the intermediate indole-2-hydrazide was collected by filtration. To a solution of indole-2-hydrazide (175 mg, 1 mmol) in 10 mL absolute ethanol were added carbon disulfide (0.3 mL, 5 mmol) and potassium hydroxide (84 mg, 1.5 mmol) and the mixture was then heated under reflux for 48 h. After concentration, the residue was diluted with water and acidified with 2N HCl solution. The product was obtained by filtration and recrystallization from methanol.

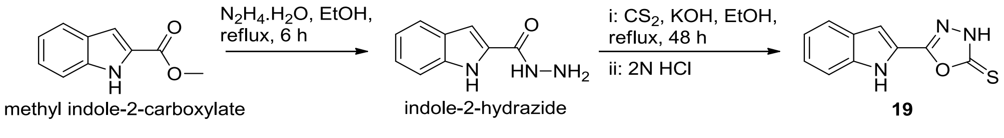

2.4 Synthesis of compound 21 from indole with chloroacetyl chloride was reference to the literature method [2].

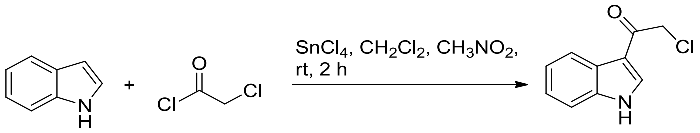

2.5 Synthesis of compound 28. To a solution of indole (117 mg, 1 mmol) in 10 mL N,N-dimethylformamide (DMF) were added sodium hydride (44 mg, 1.1 mmol) at 0 ^{o}C. After stirred at 0 ^{o}C for 30 min, 2-chloro-5-chloromethylpyridine (178 mg, 1.1 mmol) was added and the reaction mixture were stirred at room temperature for 2 h. Then the reaction mixture was diluted with water, extracted with ethyl acetate (3×15 mL). The extracts were combined, washed with brine, dried over $MgSO_4$, filtered and concentrated in vacuo. The residue was then purified by flash chromatography on silica gel with a mixture eluent of petroleum ether and ethyl acetate to give the desired compound **28**.

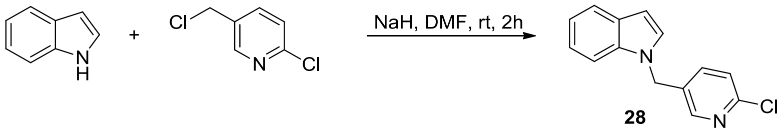

2.6 Synthesis of compounds 29~46 [3]. To a solution of indole (117 mg, 1 mmol) in 20 mL dichloromethane were added tetrabutylammonium hydrogen sulfate (34 mg, 0.1 mmol) and freshly powdered NaOH (200 mg, 5 mmol). After being stirred for 15 min, various acyl chloride (3 mmol) was added and the reaction mixture was vigorously stirred for 2 h, diluted with water and extracted with dichloromethane (3×15 mL). The extracts were combined, washed with brine, dried over $MgSO_4$, filtered and concentrated in vacuo. The residue was then purified by flash chromatography on silica gel with a mixture eluent of petroleum ether and ethyl acetate to give the desired compound **29~46**.

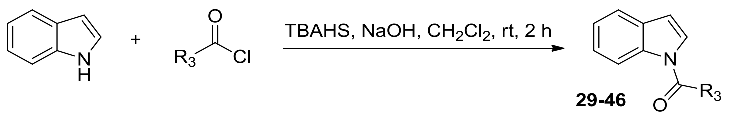

2.7 Synthesis of compound 47. The compound was synthesized with indole and 4-methylbenzene-1-sulfonyl chloride using a similar procedure as for compound **28**.

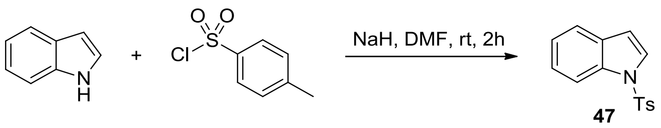

2.8 Synthesis of compounds 48~52. The compounds were synthesized from indole with various chloroformates or 4-chlorophenyl isocyanate using a similar procedure as for compound **28**.

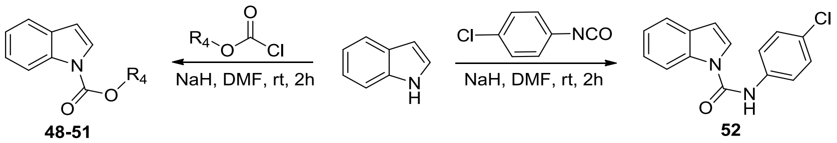

2.9 Synthesis of compounds 55, 56, 58, 60 and 74 from substituted 2-nitrotoluene with DMFDMA was reference to the literature method [4].

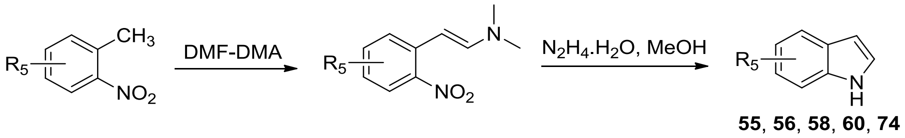

2.10 Synthesis of compounds 80~87. The compounds were prepared from various aza-indoles or substituted indole with 2-chlorobenzoyl chloride using a similar procedure as for compound **29**.

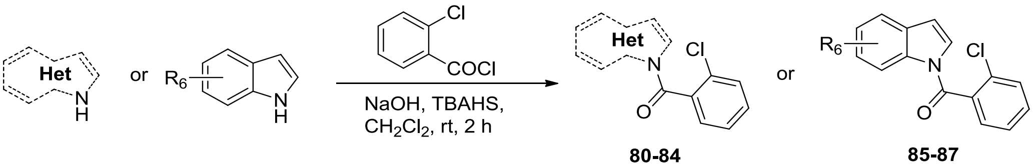

3. Characterization of Products and intermediates

***N*-cyclopropyl-1*H*-indole-2-carboxamide (11)**, white solid, yield: 88%; ^{1}H

NMR (600 MHz, $CDCl_3$) δ: 9.27 (s, 1H, 1-NH), 7.64 (d, J = 8.0 Hz, 1H, 7-H), 7.44 (d, J = 8.3 Hz, 1H, 4-H), 7.29 (t, J = 7.6 Hz, 1H, 6-H), 7.14 (t, J = 7.5 Hz, 1H, 5-H), 6.77 (s, 1H, 3-H), 6.30 (s, 1H, NH), 2.95-2.88 (m, 1H, CH), 0.91 (t, J = 6.3 Hz, 2H, CH_2), 0.68 (s, 2H, CH_2).

***N*-morpholino-1*H*-indole-2-carboxamide (12)**, white solid, yield: 83%; ^{1}H NMR (600 MHz, $CDCl_3$) δ: 9.26 (s, 1H, 1-NH), 7.65 (d, J = 8.0 Hz, 1H, 7-H), 7.43 (d, J = 8.2 Hz, 1H, 4-H), 7.30 (t, J = 7.6 Hz, 1H, 6-H), 7.15 (t, J = 7.5 Hz, 1H, 5-H), 6.78 (s, 1H, 3-H), 3.96 (s, 4H, CH_2), 3.79 (d, J = 4.2 Hz, 4H, CH_2).

***N*-[2-(morpholine-4-yl)ethyl]-1*H*-indole-2-carboxamide (13)**, white solid, yield: 91%; ^{1}H NMR (600 MHz, $CDCl_3$) δ: 9.18 (s, 1H, 1-NH), 7.67 (d, J = 8.1 Hz, 1H, 7-H), 7.44 (d, J = 8.4 Hz, 1H, 4-H), 7.29 (t, J = 7.6 Hz, 1H, 6-H), 7.15 (t, J = 7.5 Hz, 1H, 5-H), 6.85 (s, 2H, 3-H, NH), 3.77 (s, 4H, CH_2), 3.58 (dd, J = 11.1, 5.5 Hz, 2H, CH_2), 2.62 (t, J = 5.9 Hz, 2H, CH_2), 2.53 (s, 4H, CH_2).

***N*-(4-chlorophenyl)-1*H*-indole-2-carboxamide (14)**, white solid, yield: 80%; ^{1}H NMR (600 MHz, $CDCl_3$) δ: 9.33 (s, 1H, NH), 7.84 (s, 1H, NH), 7.72 (d, J = 8.2 Hz, 1H, 7-H), 7.64 (t, J = 9.2 Hz, 2H, Ar-H), 7.49 (d, J = 8.2 Hz, 1H, 4-H), 7.41-7.34 (m, 3H, 6-H, Ar-H), 7.20 (t, J = 7.5 Hz, 1H, 5-H), 7.03 (s, 1H, 3-H).

Methyl 1*H*-indol-2-ylcarbamate (15), white solid, yield: 65%; ^{1}H NMR (600 MHz, $CDCl_3$) δ: 9.82 (s, 1H, 1-NH), 7.45 (d, J = 7.0 Hz, 1H, 7-H), 7.31 (d, J = 7.3 Hz, 1H, 4-H), 7.16 (s, 1H, 3-H), 7.12-7.06 (m, 2H, 5,6-H), 5.83 (s, 1H, NH), 3.84 (s, 3H, CH_3).

Ethyl 1*H*-indol-2-ylcarbamate (16), white solid, yield: 58%; ^{1}H NMR (600

MHz, $CDCl_3$) *δ*: 9.84 (s, 1H, 1-NH), 7.47-7.41 (m, 1H, 7-H), 7.30 (d, *J* = 7.2 Hz, 1H, 4-H), 7.12-7.04 (m, 3H, 3, 5, 6-H), 5.81 (s, 1H, NH), 4.28 (dd, *J* = 14.2, 7.1 Hz, 2H, $O\underline{CH_2}CH_3$), 1.34 (t, *J* = 7.1 Hz, 3H, $OCH_2\underline{CH_3}$).

***Iso*-propyl 1*H*-indol-2-ylcarbamate (17)**, white solid, yield: 68%; ^{1}H NMR (600 MHz, $CDCl_3$) *δ*: 9.87 (s, 1H, 1-NH), 7.48-7.41 (m, 1H, 7-H), 7.32-7.26 (m, 1H, 4-H), 7.08 (dq, *J* = 7.0, 5.6 Hz, 3H, 3, 5, 6-H), 5.80 (s, 1H, NH), 5.05 (dt, *J* = 12.4, 6.1 Hz, 1H, CH), 1.33 (d, *J* = 6.2 Hz, 6H, CH_3).

Butyl 1*H*-indol-2-ylcarbamate (18), white solid, yield: 53%; ^{1}H NMR (600 MHz, $CDCl_3$) *δ*: 9.85 (s, 1H, 1-NH), 7.47-7.42 (m, 1H, 7-H), 7.30 (d, *J* = 6.9 Hz, 1H, 4-H), 7.14 (s, 1H, 3-H), 7.12-7.05 (m, 2H, 5, 6-H), 5.81 (s, 1H, NH), 4.22 (t, *J* = 6.6 Hz, 2H, $O\underline{CH_2}$-), 1.68 (dd, *J* = 14.5, 7.0 Hz, 2H, 2'-CH_2), 1.43 (dq, *J* = 14.9, 7.5 Hz, 2H, 3'-CH_2), 0.97 (t, *J* = 7.4 Hz, 3H, 2'-CH_3).

2-(2-thioxo-1,3,4-oxadiazol-5-yl)-1*H*-indole (19), white solid, yield: 83%; ^{1}H NMR (600 MHz, $CDCl_3$) *δ*: 14.78 (brs, 1H, NH), 12.20 (s, 1H, 1-NH), 7.67 (d, *J* = 8.0 Hz, 1H, 7-H), 7.45 (dd, *J* = 8.3, 0.7 Hz, 1H, 4-H), 7.28-7.25 (m, 1H, 6-H), 7.18 (d, *J* = 1.4 Hz, 1H, 5-H), 7.13-7.08 (m, 1H, 3-H).

3-chloroacetyl-1*H*-indole (21), light brown solid, yield: 52%; ^{1}H NMR (600 MHz, $CDCl_3$) *δ*: 12.12 (s, 1H, 1-NH), 8.44 (d, *J* = 3.2 Hz, 1H, 4-H), 8.16 (d, *J* = 7.6 Hz, 1H, 7-H), 7.50 (d, *J* = 7.5 Hz, 1H, 2-H), 7.23 (tt, *J* = 14.5, 7.1 Hz, 2H, 5, 6-H), 4.88 (s, 2H, -CH_2Cl).

1-(6-chloro-3-pyridylmethyl)-1*H*-indole (28), white solid, yield: 67%; ^{1}H NMR (600 MHz, $CDCl_3$) *δ*: 8.29 (d, *J* = 2.2 Hz, 1H, Ar-H), 7.66 (d, *J* = 7.9 Hz, 1H,

4-H), 7.25-7.18 (m, 4H, 6, 7-H, Ar-H), 7.14 (t, *J* = 7.3 Hz, 1H, 5-H), 7.11 (d, *J* = 3.2 Hz, 1H, 2-H), 6.59 (d, *J* = 3.2 Hz, 1H, 3-H), 5.32 (s, 2H, CH_2).

1-(1-oxopropyl)-1*H*-indole (30), white solid, yield: 88%; ^{1}H NMR (600 MHz, $CDCl_3$) δ: 8.47 (d, *J* = 8.1 Hz, 1H, 7-H), 7.57 (d, *J* = 7.7 Hz, 1H, 4-H), 7.47 (d, *J* = 3.4 Hz, 1H, 2-H), 7.36 (t, *J* = 7.7 Hz, 1H, 6-H), 7.28 (d, *J* = 7.6 Hz, 1H, 5-H), 6.64 (d, *J* = 3.7 Hz, 1H, 3-H), 2.97 (q, *J* = 7.3 Hz, 2H, CH_2), 1.35 (t, *J* = 7.3 Hz, 3H, CH_3).

1-(1-oxobutyl)-1*H*-indole (31), white solid, yield: 83%; ^{1}H NMR (600 MHz, $CDCl_3$) δ: 8.48 (d, *J* = 8.1 Hz, 1H, 7-H), 7.57 (d, *J* = 7.7 Hz, 1H, 4-H), 7.47 (d, *J* = 3.6 Hz, 1H, 2-H), 7.36 (dd, *J* = 11.4, 4.1 Hz, 1H, 6-H), 7.30-7.26 (m, 1H, 5-H), 6.64 (d, *J* = 3.7 Hz, 1H, 3-H), 2.90 (t, *J* = 7.4 Hz, 2H, -CO$\underline{CH_2}$-), 1.92-1.84 (m, 2H, 3'-CH_2), 1.08 (t, *J* = 7.4 Hz, 3H, 4'-CH_3).

1-(1-oxopentyl)-1*H*-indole (32), colorless oil, yield: 79%; ^{1}H NMR (600 MHz, $CDCl_3$) δ: 8.48 (d, *J* = 8.1 Hz, 1H, 7-H), 7.57 (d, *J* = 7.7 Hz, 1H, 4-H), 7.47 (d, *J* = 3.6 Hz, 1H, 2-H), 7.37-7.33 (m, 2H, 5, 6-H), 6.64 (d, *J* = 3.7 Hz, 1H, 3-H), 2.92 (t, *J* = 7.5 Hz, 2H, -CO$\underline{CH_2}$-), 1.86-1.79 (m, 2H, 3'-CH_2), 1.52-1.44 (m, 2H, 4'-CH_2), 0.99 (t, *J* = 7.4 Hz, 3H, 5'-CH_3).

1-(1-oxohexyl)-1*H*-indole (33), colorless oil, yield: 74%; ^{1}H NMR (600 MHz, $CDCl_3$) δ: 8.48 (d, *J* = 8.0 Hz, 1H, 7-H), 7.57 (d, *J* = 7.7 Hz, 1H, 4-H), 7.47 (d, *J* = 3.5 Hz, 1H, 2-H), 7.36 (dd, *J* = 11.4, 4.1 Hz, 1H, 6-H), 7.29-7.27 (m, 1H, 5-H), 6.64 (d, *J* = 3.6 Hz, 1H, 3-H), 2.91 (t, *J* = 7.5 Hz, 2H, -CO$\underline{CH_2}$-), 1.88-1.81 (m, 2H, 3'-CH_2), 1.46-1.36 (m, 4H, 4', 5'-CH_2), 0.94 (t, *J* = 7.1 Hz, 3H, 6'-CH_3).

1-(1-oxoheptyl)-1*H*-indole (34), colorless oil, yield: 71%; ^{1}H NMR (600 MHz,

$CDCl_3$) δ: 8.48 (d, *J* = 8.1 Hz, 1H, 7-H), 7.57 (d, *J* = 7.7 Hz, 1H, 4-H), 7.47 (d, *J* = 3.5 Hz, 1H, 2-H), 7.35 (t, *J* = 7.4 Hz, 1H, 6-H), 7.28 (d, *J* = 7.3 Hz, 1H, 5-H), 6.64 (d, *J* = 3.7 Hz, 1H, 3-H), 2.91 (t, *J* = 7.5 Hz, 2H, -CO$\underline{CH_2}$-), 1.87-1.79 (m, 2H, 3'-CH_2), 1.44 (dd, *J* = 14.8, 6.9 Hz, 2H, 4'-CH_2), 1.38-1.31 (m, 4H, 5', 6'-CH_2), 0.91 (t, *J* = 6.9 Hz, 3H, 7'-CH_3).

1-cyclopropylcarbonyl-1*H*-indole (35), white solid, yield: 82%; ^{1}H NMR (600 MHz, $CDCl_3$) δ: 8.43 (d, *J* = 8.3 Hz, 1H, 7-H), 7.71 (d, *J* = 3.7 Hz, 1H, 4-H), 7.58 (d, *J* = 7.7 Hz, 1H, 2-H), 7.34 (dd, *J* = 11.4, 4.1 Hz, 1H, 6-H), 7.29-7.26 (m, 1H, 5-H), 6.68 (d, *J* = 3.6 Hz, 1H, 3-H), 2.33-2.26 (m, 1H, -CH-), 1.35-1.31 (m, 2H), 1.12-1.06 (m, 2H).

1-cyclobutylcarbonyl-1*H*-indole (36), white solid, yield: 79%; ^{1}H NMR (600 MHz, $CDCl_3$) δ: 8.50 (d, *J* = 8.2 Hz, 1H, 7-H), 7.56 (d, *J* = 7.7 Hz, 1H, 4-H), 7.36 (t, *J* = 7.4 Hz, 1H, 6-H), 7.32 (d, *J* = 3.4 Hz, 1H, 2-H), 7.28 (d, *J* = 7.2 Hz, 1H, 5-H), 6.62 (d, *J* = 3.7 Hz, 1H, 3-H), 3.83 (p, *J* = 8.4 Hz, 1H, -CH-), 2.57 (dt, *J* = 18.0, 9.0 Hz, 2H, CH_2), 2.41-2.34 (m, 2H, CH_2), 2.17-2.07 (m, 1H, CH), 2.04-1.96 (m, 1H, CH).

1-cyclopentylcarbonyl-1*H*-indole (37), light yellow oil, yield: 73%; ^{1}H NMR (600 MHz, $CDCl_3$) δ: 8.50 (d, *J* = 8.3 Hz, 1H, 7-H), 7.56 (d, *J* = 7.7 Hz, 1H, 4-H), 7.53 (d, *J* = 3.7 Hz, 1H, 2-H), 7.35 (t, *J* = 7.4 Hz, 1H, 6-H), 7.29-7.26 (m, 1H, 5-H), 6.64 (d, *J* = 3.7 Hz, 1H, 3-H), 3.50-3.44 (m, 1H, -CH-), 2.09-2.03 (m, 2H, CH_2), 1.82 (dd, *J* = 9.3, 5.8 Hz, 2H, CH_2), 1.73-1.67 (m, 4H, CH_2).

1-cyclohexylcarbonyl-1*H*-indole (38), light yellow oil, yield: 78%; ^{1}H NMR (600 MHz, $CDCl_3$) δ: 8.49 (d, *J* = 8.3 Hz, 1H, 7-H), 7.56 (d, *J* = 7.7 Hz, 1H, 4-H),

7.51 (d, *J* = 3.7 Hz, 1H, 2-H), 7.35 (t, *J* = 7.6 Hz, 1H, 6-H), 7.28 (d, *J* = 7.6 Hz, 1H, 5-H), 6.65 (d, *J* = 3.7 Hz, 1H, 3-H), 3.01 (ddd, *J* = 11.6, 8.3, 3.3 Hz, 1H, -CH-), 2.01 (d, *J* = 12.8 Hz, 2H, CH_2), 1.90 (d, *J* = 13.3 Hz, 2H, CH_2), 1.76 (dd, *J* = 12.2, 6.8 Hz, 2H, CH_2), 1.69-1.64 (m, 2H, CH_2), 1.43 (dt, *J* = 25.9, 9.1 Hz, 2H, CH_2).

1-benzoyl-1*H*-indole (39), white solid, yield: 76%; ^{1}H NMR (600 MHz, $CDCl_3$) δ: 8.54 (brs, 1H, 7-H), 7.86-7.82 (m, 1H, Ar-H), 7.72-7.67 (m, 2H, Ar-H), 7.59 (d, *J* = 7.9 Hz, 1H, 4-H), 7.57-7.52 (m, 1H, 2-H), 7.40 (s, 1H, Ar-H), 7.34 (t, *J* = 7.4 Hz, 1H, 6-H), 6.84 (brs, 1H, 5-H), 6.59 (d, *J* = 3.2 Hz, 1H, 3-H).

1-(2-fluorobenzoyl)-1*H*-indole (40), white solid, yield: 85%; ^{1}H NMR (600 MHz, $CDCl_3$) δ: 8.45 (d, *J* = 7.5 Hz, 1H, 7-H), 7.61-7.55 (m, 3H, 4-H, Ar-H), 7.40 (t, *J* = 7.7 Hz, 1H, Ar-H), 7.33 (td, *J* = 7.0, 4.5 Hz, 2H, 5, 6-H), 7.23 (t, *J* = 9.0 Hz, 1H, Ar-H), 7.14-7.10 (m, 1H, Ar-H), 6.62 (d, *J* = 3.8 Hz, 1H, 3-H).

1-(2-chlorobenzoyl)-1*H*-indole (41), white solid, yield: 83%; ^{1}H NMR (600 MHz, $CDCl_3$) δ: 8.46 (brs, 1H, 7-H), 7.59 (d, *J* = 7.7 Hz, 1H, 4-H), 7.55-7.48 (m, 3H, 2-H, Ar-H), 7.45-7.37 (m, 2H, 6-H, Ar-H), 7.33 (t, *J* = 7.5 Hz, 1H, Ar-H), 6.97 (brs, 1H, 5-H), 6.61 (d, *J* = 3.7 Hz, 1H, 3-H).

1-(2-bromobenzoyl)-1*H*-indole (42), white solid, yield: 77%; ^{1}H NMR (600 MHz, $CDCl_3$) δ: 8.46 (brs, 1H, 7-H), 7.70 (d, *J* = 8.0 Hz, 1H, Ar-H), 7.59 (d, *J* = 7.8 Hz, 1H, 4-H), 7.50-7.46 (m, 2H, 2-H, Ar-H), 7.44-7.38 (m, 2H, Ar-H), 7.33 (t, *J* = 7.5 Hz, 1H, 6-H), 6.96 (brs, 1H, 5-H), 6.61 (d, *J* = 3.7 Hz, 1H, 3-H).

1-(2-trifluoromethylbenzoyl)-1*H*-indole (43), white solid, yield: 80%; ^{1}H NMR (600 MHz, DMSO-d_6) δ: 8.30 (s, 1H, 7-H), 7.99 (d, *J* = 7.5 Hz, 1H, Ar-H), 7.91-7.83

(m, 3H, 4-H, Ar-H), 7.68 (d, *J* = 7.7 Hz, 1H, 2-H), 7.39 (d, *J* = 7.3 Hz, 1H, Ar-H), 7.35 (t, *J* = 7.4 Hz, 1H, 6-H), 7.08 (s, 1H, 5-H), 6.75 (d, *J* = 3.6 Hz, 1H, 3-H).

1-(3-trifluoromethylbenzoyl)-1*H*-indole (44), white solid, yield: 71%; ^{1}H NMR (600 MHz, $CDCl_3$) *δ*: 8.41 (d, *J* = 8.2 Hz, 1H, 7-H), 8.02 (s, 1H, Ar-H), 7.93 (d, *J* = 7.7 Hz, 1H, Ar-H), 7.88 (d, *J* = 7.9 Hz, 1H, 4-H), 7.69 (t, *J* = 7.8 Hz, 1H, Ar-H), 7.62 (d, *J* = 7.7 Hz, 1H, 2-H), 7.43-7.40 (m, 1H, Ar-H), 7.37-7.33 (m, 1H, 6-H), 7.20 (d, *J* = 3.8 Hz, 1H, 5-H), 6.67 (d, *J* = 3.7 Hz, 1H, 3-H).

1-(4-trifluoromethylbenzoyl)-1*H*-indole (45), white solid, yield: 77%; ^{1}H NMR (600 MHz, $CDCl_3$) *δ*: 8.42 (d, *J* = 8.3 Hz, 1H, 7-H), 8.28 (d, *J* = 8.1 Hz, 1H, 4-H), 7.86 (d, *J* = 8.1 Hz, 2H, Ar-H), 7.82 (t, *J* = 7.2 Hz, 2H, Ar-H), 7.62 (d, *J* = 7.7 Hz, 1H, 2-H), 7.44-7.39 (m, 1H, 6-H), 7.37-7.32 (m, 1H, 5-H), 7.19 (d, *J* = 3.8 Hz, 1H, Ar-H), 6.65 (d, *J* = 3.7 Hz, 1H, 3-H).

1-(2-trifluoromethoxybenzoyl)-1*H*-indole (46), white solid, yield: 82%; ^{1}H NMR (600 MHz, $CDCl_3$) *δ*: 8.40 (d, *J* = 8.2 Hz, 1H, 7-H), 7.82-7.79 (m, 2H, Ar-H), 7.62 (d, *J* = 7.7 Hz, 1H, 4-H), 7.43-7.36 (m, 3H, 2-H, Ar-H), 7.35-7.32 (m, 1H, 6-H), 7.25 (s, 1H, 5-H), 6.65 (d, *J* = 3.7 Hz, 1H, 3-H).

1-tosyl-1*H*-indole (47), white solid, yield: 88%; ^{1}H NMR (600 MHz, $CDCl_3$) *δ*: 7.99 (d, *J* = 8.3 Hz, 1H, 7-H), 7.76 (d, *J* = 8.4 Hz, 2H, Ar-H), 7.56 (d, *J* = 3.7 Hz, 1H, 4-H), 7.52 (d, *J* = 7.8 Hz, 1H, 2-H), 7.30 (t, *J* = 7.7 Hz, 1H, 6-H), 7.22 (t, *J* = 7.3 Hz, 3H, 5-H, Ar-H), 6.65 (d, *J* = 3.6 Hz, 1H, 3-H), 2.33 (s, 3H, CH_3).

Methyl 1*H*-indole-1-carboxylate (48), colorless oil, yield: 68%; ^{1}H NMR (600 MHz, $CDCl_3$) *δ*: 8.20 (brs, 1H, 7-H), 7.61 (s, 1H, 4-H), 7.58 (d, *J* = 7.8 Hz, 1H, 2-H),

7.35 (t, J = 7.6 Hz, 1H, 6-H), 7.26 (dd, J = 10.9, 4.1 Hz, 1H, 5-H), 6.61 (d, J = 3.6 Hz, 1H, 3-H), 4.05 (s, 3H, CH_3).

Ethyl 1*H*-indole-1-carboxylate (49), colorless oil, yield: 73%; ^{1}H NMR (600 MHz, $CDCl_3$) δ: 8.19 (d, J = 6.0 Hz, 1H, 7-H), 7.63 (d, J = 3.5 Hz, 1H, 4-H), 7.57 (d, J = 7.8 Hz, 1H, 2-H), 7.36-7.31 (m, 1H, 6-H), 7.25-7.22 (m, 1H, 5-H), 6.60 (d, J = 3.6 Hz, 1H, 3-H), 4.50 (q, J = 7.1 Hz, 2H, CH_2), 1.48 (t, J = 7.1 Hz, 3H, CH_3).

***Iso*-propyl 1*H*-indole-1-carboxylate (50)**, colorless oil, yield: 78%; ^{1}H NMR (600 MHz, $CDCl_3$) δ: 8.18 (s, 1H, 7-H), 7.63 (s, 1H, 4-H), 7.57 (d, J = 7.8 Hz, 1H, 2-H), 7.33 (t, J = 7.7 Hz, 1H, 6-H), 7.26-7.23 (m, 1H, 5-H), 6.60 (d, J = 3.6 Hz, 1H, 3-H), 5.28 (dt, J = 12.5, 6.3 Hz, 1H, -CH-), 1.46 (d, J = 6.2 Hz, 6H, CH_3).

***Tert*-butyl 1*H*-indole-1-carboxylate (51)**, colorless oil, yield: 88%; ^{1}H NMR (600 MHz, $CDCl_3$) δ: 8.14 (d, J = 6.5 Hz, 1H, 7-H), 7.60 (d, J = 3.1 Hz, 1H, 4-H), 7.56 (d, J = 7.8 Hz, 1H, 2-H), 7.31 (t, J = 7.7 Hz, 1H, 6-H), 7.22 (t, J = 7.5 Hz, 1H, 5-H), 6.57 (d, J = 3.7 Hz, 1H, 3-H), 1.68 (s, 9H, CH_3).

***N*-(4-chlorophenyl)-1*H*-indol-1-carboxamide (52)**, white solid, yield: 68%; ^{1}H NMR (600 MHz, $CDCl_3$) δ: 8.10 (d, J = 8.3 Hz, 1H, 7-H), 7.64 (d, J = 7.8 Hz, 1H, 4-H), 7.54 (d, J = 3.7 Hz, 1H, 2-H), 7.52-7.48 (m, 2H, Ar-H), 7.38-7.35 (m, 3H, 6-H, Ar-H), 7.29-7.27 (m, 1H, 5-H), 6.71 (d, J = 3.6 Hz, 1H, 3-H).

4-fluoro-1*H*-indole (55), white solid, yield: 68%; ^{1}H NMR (600 MHz, $CDCl_3$) δ: 8.24 (brs, 1H, NH), 7.19 (dd, J = 6.3, 3.1 Hz, 2H, 7-H, 2-H), 7.11 (td, J = 8.0, 5.1 Hz, 1H, 6-H), 6.79 (dd, J = 10.2, 7.9 Hz, 1H, 5-H), 6.65 (dd, J = 3.7, 1.5 Hz, 1H, 3-H).

4-chloro-1*H*-indole (56), light yellow liquid, yield: 75%; ^{1}H NMR (600 MHz,

$CDCl_3$) δ: 8.27 (s, 1H, NH), 7.30 (ddd, J = 7.1, 1.6, 0.9 Hz, 1H, 7-H), 7.25 (d, J = 2.9 Hz, 1H, 2-H), 7.14-7.09 (m, 2H, 5-H, 6-H), 6.68-6.66 (m, 1H, 3-H).

4-nitro-1*H*-indole (58), yellow powder, yield: 62%; ^{1}H NMR (600 MHz, $CDCl_3$) δ: 8.61 (brs, 1H, 1-NH), 8.16 (dd, J = 8.0, 0.5 Hz, 1H, 7-H), 7.73 (d, J = 8.0 Hz, 1H, 2-H), 7.49 (t, J = 2.9 Hz, 1H, 6-H), 7.34-7.32 (m, 1H, 5-H), 7.29 (t, J = 8.0 Hz, 1H, 3-H).

4-trifluoromethyl-1*H*-indole (60), white powder, yield: 42%; ^{1}H NMR (600 MHz, $CDCl_3$) δ: 8.37 (brs, 1H, NH), 7.57 (d, J = 8.1 Hz, 1H, 7-H), 7.43 (d, J = 7.4 Hz, 1H, 2-H), 7.35-7.32 (m, 1H, 6-H), 7.26 (t, J = 7.8 Hz, 1H, 5-H), 6.76 (d, J = 1.6 Hz, 1H, 3-H).

5-acetyl-1*H*-indole (74), white solid, yield: 53%; ^{1}H NMR (600 MHz, $CDCl_3$) δ: 8.48 (brs, 1H, NH), 8.33 (s, 1H, 4-H), 7.89 (dd, J = 8.6, 1.5 Hz, 1H, 7-H), 7.42 (d, J = 8.6 Hz, 1H, 2-H), 7.29 (t, J = 2.8 Hz, 1H, 6-H), 6.68 (s, 1H, 3-H), 2.67 (s, 3H, $COCH_3$).

1-(2-chlorobenzoyl)-1*H*-indazole (80), white solid, yield: 71%; ^{1}H NMR (600 MHz, $CDCl_3$) δ: 8.59 (d, J = 8.3 Hz, 1H, 7-H), 8.17 (s, 1H, 3-H), 7.78 (d, J = 7.9 Hz, 1H, Ar-H), 7.68-7.64 (m, 1H, 4-H), 7.56 (dd, J = 7.5, 1.5 Hz, 1H, 6-H), 7.50 (ddd, J = 11.2, 7.6, 1.5 Hz, 2H, Ar-H), 7.47-7.40 (m, 2H, 5-H, Ar-H).

1-(2-chlorobenzoyl)-1*H*-benzimidazole (81), white solid, yield: 77%; ^{1}H NMR (600 MHz, $CDCl_3$) δ: 8.21 (d, J = 5.8 Hz, 1H, 7-H), 7.92 (s, 1H, 2-H), 7.85-7.82 (m, 1H, Ar-H), 7.60-7.56 (m, 3H, 4,6-H, Ar-H), 7.51-7.44 (m, 3H, 5-H, Ar-H).

1-(2-chlorobenzoyl)-5-Aza-1*H*-indole (82), white solid, yield: 82%; ^{1}H NMR

(600 MHz, $CDCl_3$) δ: 8.95 (d, J = 0.7 Hz, 1H, 4-H), 8.56 (d, J = 5.6 Hz, 1H, 6-H), 8.14 (s, 1H, 7-H), 7.56-7.50 (m, 3H, Ar-H), 7.49-7.43 (m, 1H, Ar-H), 7.10 (s, 1H, 2-H), 6.70 (d, J = 3.7 Hz, 1H, 3-H).

1-(2-chlorobenzoyl)-6-Aza-1*H*-indole (83), white solid, yield: 85%; ^{1}H NMR (600 MHz, $CDCl_3$) δ: 9.62 (brs, 1H, 7-H), 8.51 (d, J = 5.1 Hz, 1H, 5-H), 7.54 (t, J = 5.2 Hz, 4H, Ar-H), 7.50 – 7.44 (m, 1H, 4-H), 7.18 (brs, 1H, 2-H), 6.63 (d, J = 3.6 Hz, 1H, 3-H).

1-(2-chlorobenzoyl)-7-Aza-1*H*-indole (84), white solid, yield: 74%; ^{1}H NMR (600 MHz, $CDCl_3$) δ: 8.21 (dd, J = 4.8, 1.4 Hz, 1H, 4-H), 7.87 (dd, J = 7.8, 1.6 Hz, 1H, 6-H), 7.70 (d, J = 4.1 Hz, 1H, Ar-H), 7.52 (dd, J = 7.5, 1.4 Hz, 1H, Ar-H), 7.49-7.45 (m, 2H, Ar-H), 7.41 (td, J = 7.3, 1.6 Hz, 1H, 5-H), 7.16 (dd, J = 7.8, 4.8 Hz, 1H, 2-H), 6.65 (d, J = 4.1 Hz, 1H, 3-H).

1-(2-chlorobenzoyl)-4-fluoro-1*H*-indole (85), white solid, yield: 78%; ^{1}H NMR (600 MHz, $CDCl_3$) δ: 8.20 (brs, 1H, 7-H), 7.55-7.49 (m, 3H, Ar-H), 7.46-7.42 (m, 1H, Ar-H), 7.33 (dt, J = 13.6, 6.9 Hz, 1H, 2-H), 7.05-7.00 (m, 1H, 6-H), 6.96 (s, 1H, 5-H), 6.71 (d, J = 3.8 Hz, 1H, 3-H).

1-(2-chlorobenzoyl)-4-chloro-1*H*-indole (86), white solid, yield: 81%; ^{1}H NMR (600 MHz, $CDCl_3$) δ: 8.34 (s, 1H, 7-H), 7.53-7.50 (m, 3H, Ar-H), 7.46-7.42 (m, 1H, Ar-H), 7.34-7.31 (m, 2H, 2-H, 6-H), 7.02 (s, 1H, 5-H), 6.73 (d, J = 3.8 Hz, 1H, 3-H).

1-(2-chlorobenzoyl)-5-chloro-1*H*-indole (87), white solid, yield: 84%; ^{1}H NMR (600 MHz, $CDCl_3$) δ: 8.37 (s, 1H, 7-H), 7.56 (d, J = 2.0 Hz, 1H, 4-H), 7.54-7.49 (m, 3H, Ar-H), 7.46-7.41 (m, 1H, Ar-H), 7.35 (d, J = 8.1 Hz, 1H, 2-H), 7.00 (s, 1H, 6-H),

6.55 (d, *J* = 3.7 Hz, 1H, 3-H).

REFERENCES

1. Mistry, S.N., Shonberg, J., Draperjoyce, C.J., Klein, H.C., Michino, M., Shi, L., Christopoulos, A., Capuano, B., Scammells, P.J., Lane, J.R., 2015. Discovery of a novel class of negative allosteric modulator of the dopamine D2 receptor through fragmentation of a bitopic ligand. J. Med. Chem. 58, 6819-6843.
2. Gitto, R., De Luca, L., Ferro, S., Citraro, R., De Sarro, G., Costa, L., Ciranna, L., Chimirri, A., 2009. Development of 3-substituted-1H-indole derivatives as NR2B/NMDA receptor antagonists. Bioorg. Med. Chem. 17, 1640-1647.
3. Tomakinian, T., Guillot, R., Kouklovsky, C., Vincent, G., 2014. Direct oxidative coupling of N-acetyl indoles and phenols for the synthesis of benzofuroindolines related to phalarine. Angew Chem. Int. Edit. 53, 11881-11885.
4. Batcho, A.D., Leimgruber, W., 1985. Indoles from 2-methylnitrobenzenes by condensation with formamide acetals followed by reduction: 4-benzyloxyindole. Organic Syntheses 63, 214

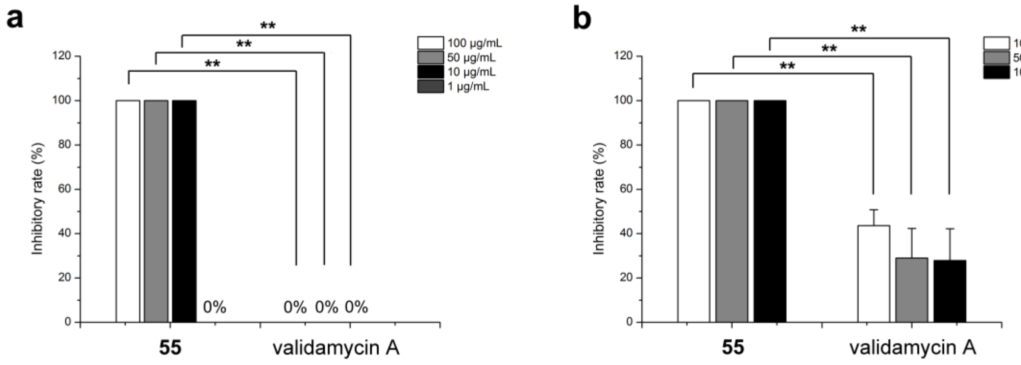

Figure S1. The inhibitory effect of compound **55** on the sclerotia germination (a) and formation (b) of *R. solani*. ** $P < 0.01$ compared to control group.

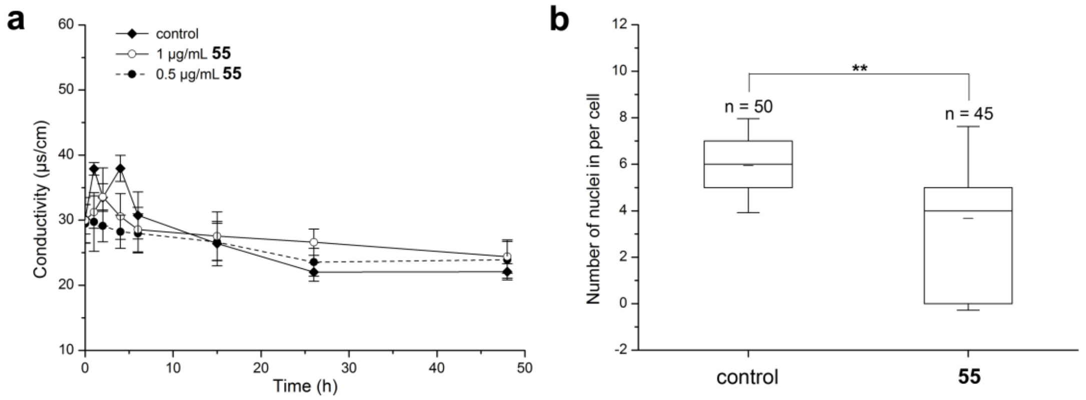

Figure S2. (a) the conductivity of the hyphae suspensions during different time exposure to **55** was measured to assess cell membrane permeability; (b) the number of nuclei in per cell of somatic hyphae of *R. solani* treated with 0 or 1 μg/mL **55**.
